# Supplementary figures and images for: QTL mapping of natural variation reveals that the developmental regulator bruno reduces tolerance to P-element transposition in the Drosophila female germline
Source: PLoS Biol. 2018 Oct 30;16(10):e2006040. doi: 10.1371/journal.pbio.2006040 (PMC6207299; doi:10.1371/journal.pbio.2006040)

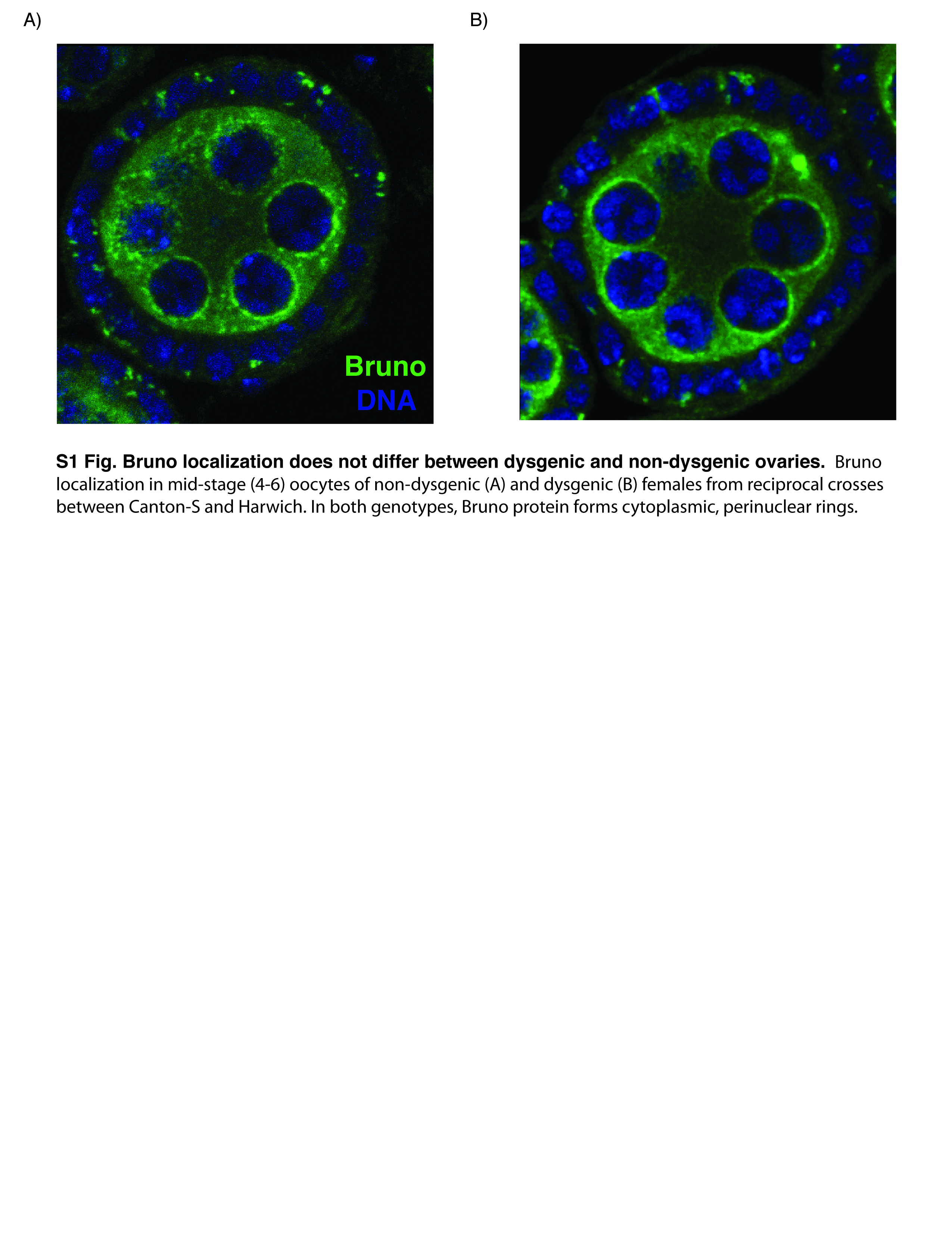

Supplement: S1 Fig — Bruno localization in mid-stage (4–6) oocytes of non-dysgenic (A) and dysgenic (B) females from reciprocal crosses between Canton-S and Harwich. In both genotypes, Bruno protein forms cytoplasmic, perinuclear rings. (TIF) [file pbio.2006040.s013.tif]

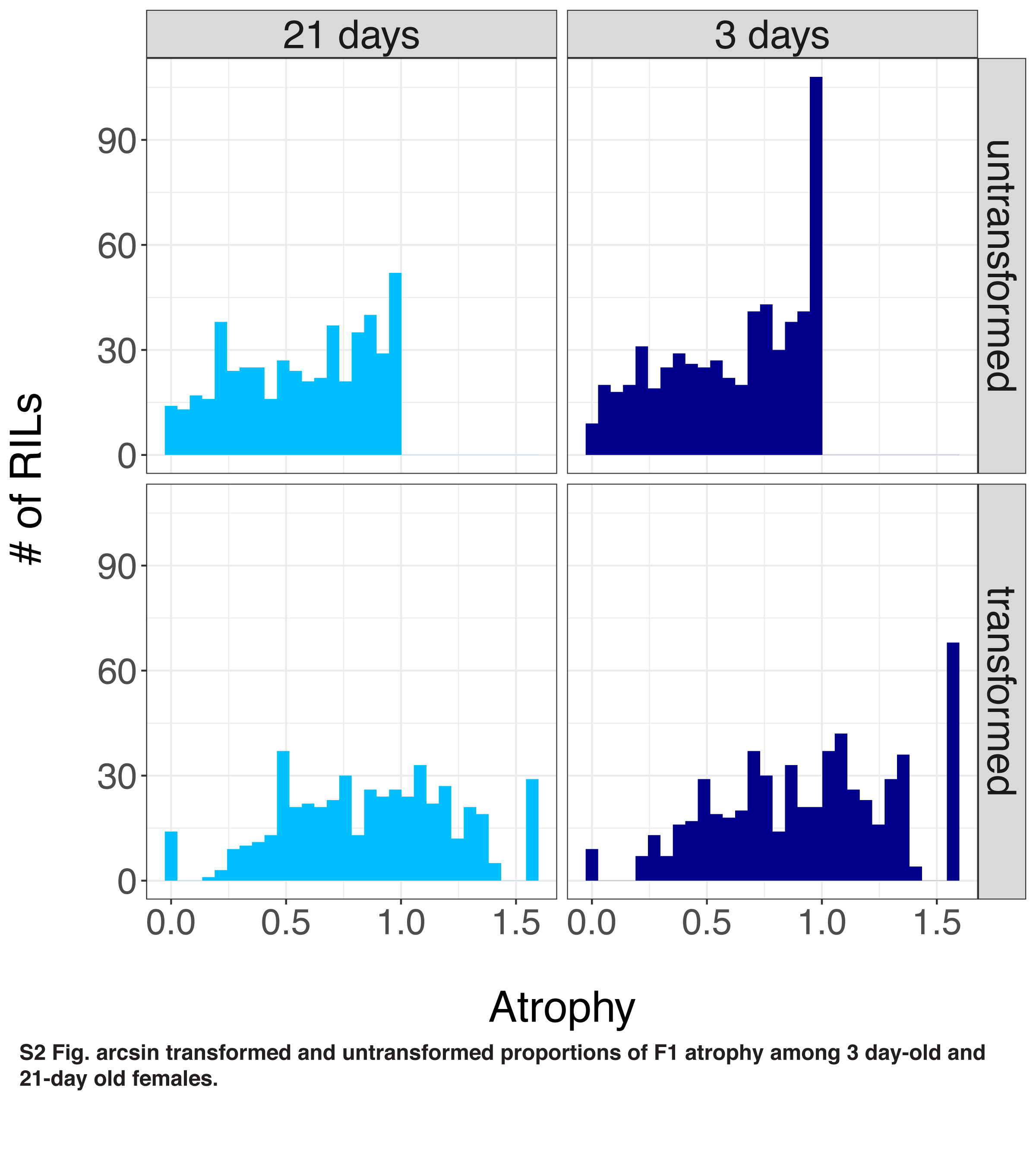

Supplement: S2 Fig — Individual data points required to generate histograms are provided in S4 and S5 Data. F1, filial 1. (TIF) [file pbio.2006040.s014.tif]
